# Editorial: Lactic Acid Bacteria: Microbial Metabolism and Expanding Applications

**DOI:** 10.3389/fbioe.2021.794164

**Published:** 2021-11-12

**Authors:** Jian-Ming Liu, Csaba Fehér, Mingfeng Cao, Fuping Lu, Peter Ruhdal Jensen

**Affiliations:** ^1^ School of Biology and Biological Engineering, South China University of Technology, Guangzhou, China; ^2^ Department of Applied Biotechnology and Food Science, Budapest University of Technology and Economics, Budapest, Hungary; ^3^ College of Chemistry and Chemical Engineering, Xiamen University, Xiamen, China; ^4^ College of Biotechnology, Tianjin University of Science and Technology, Tianjin, China; ^5^ The National Food Institute, Technical University of Denmark, Kongens Lyngby, Denmark

**Keywords:** metabolic engineering, fermentation, plant food, gut microbiome, bacteriocins, lactic acid bacteria, traditional mutagenesis

## Introduction

Lactic acid bacteria (LAB) are a group of lactic acid-forming bacteria that are generally recognized as safe (GRAS), facultatively anaerobic, non-respiring and non-sporulating. They are ubiquitous in nature, present from plant materials, milk and meat to the intestine of mammals. The metabolism of LAB is diverse and in this collection, Wang et al. reviewed LAB metabolism with a particular focus on carbon (polysaccharide) and nitrogen (protein) degradation as well as their metabolic activities to produce a large number of valuable metabolites, including organic acids, flavors, vitamins, exopolysaccharides, antimicrobial and antioxidant compounds.

## Diverse Metabolism

In LAB metabolism, one of the major features is that they produce predominantly lactic acid (LA). The LA formed as well as the drop in pH can inhibit the growth of other microorganisms and increase the shelf-life of the fermented foods (Liu et al., 2019). But the overproduction of LA, especially in non-growing conditions, might negatively affect the product flavor and quality. To measure the long-term LA production, Nugroho et al. developed a high-throughput approach to determine the metabolic pathway activities of non-growing cells, including LA production and the arginine deiminase pathway, over prolonged periods. The method was based on the real-time monitoring of pH change through the use of fluorescent pH indicators, which enabled the study of different strains and different growth conditions efficiently.

**FIGURE 1:**
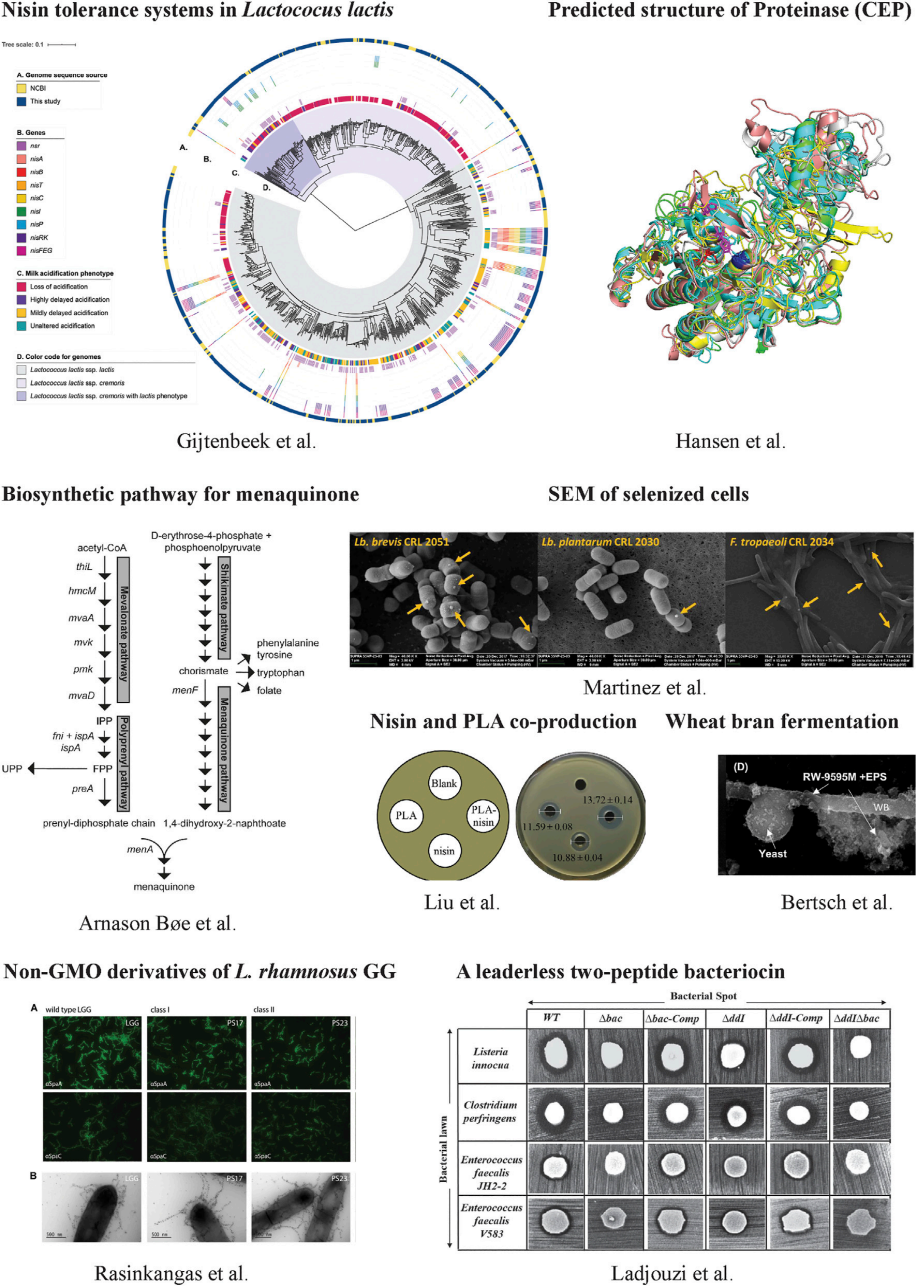
Some representative works in this collection.

Another important feature of LAB is their bacteriocins production. Bacteriocins are ribosomally synthesized antimicrobial peptides. Many bacteriocins have been used or show great potential for use in the food and clinical industry. For example, nisin is a well-established preservative against some gram-positive bacteria in foods and its production is established by a conservative biosynthetic gene cluster comprised of *nisABTCIPRK* and *nisFEG* (Kuipers et al., 1993). NisI, a membrane-associated lipoprotein, and NisFEG, an ABC transporter, confer nisin immunity. To investigate the distribution of nisin immunity not only on the producing cells but overall on dairy *Lactococcus lactis*, van Gijtenbeek et al. examined nisin tolerance on more than 700 *L. lactis* in both phenotype and genotype. They found nisin tolerance was widely distributed in non-nisin-producing *L. lactis*, contributed mainly by NSR (a nisin degradation enzyme). This work facilitates the selection of strains that do not produce nisin but have immunity for designing nisin-compatible starter cultures in food fermentations.

Nisin production is initiated by its pre-peptide synthesis with an N-terminal leader peptide, followed by the leader peptide cleavage and a typical post-translational modification step. It is different for leaderless bacteriocins that do not have any N-terminal leader sequence and can become immediately active after their translations. Enterocin DD14 is such a leaderless bacteriocin produced by *Enterococcus faecalis* 14. It shows antagonistic activities against many pathogens, such as *Staphylococcus aureus* and *Listeria monocytogenes*. It remains largely elusive how the bacteriocinogenic strain can develop the immunity for its own bacteriocin that has activities immediately after translation. Ladjouzi et al. investigated the immunity system of EntDD14 at the molecular level and demonstrated that intracellular EntDD14 is involved in self-immunity.

Bacteriocins show great potential to fight various pathogens. Todorov et al. discussed the potential to harness bacteriocins widely produced by different LAB species as promising biotherapeutic agents for controlling *Clostridium* and *Clostridiodes* infections. The pathogenic *Clostridium* and *Clostridiodes* spp. are difficult to tackle because of their ability to form highly resistant endospores and antibiotic resistance and they are directly related to food poisoning and human gastrointestinal problems. A number of bacteriocins are effective against *Clostridium* and *Clostridiodes* species.

## Expanding Applications

Some LAB are widely used in the dairy industry for yogurt and cheese (Liu et al., 2019). One important reason that these LAB can grow efficiently in the milk-derived matrix is due to their excellent proteolytic system, including proteinase, peptide transporter and peptidase, to metabolize milk proteins. Hansen and Marcatili presented a molecular model of the cell envelope proteinase (CEP) of *L. lactis*. The *L. lactis* CEP has multi-domains and this simulation work predicts functions to the domains in the context of degradation of caseins. It makes an important contribution to our understanding of dairy protein metabolism as well as LAB growth in dairy fermentations. In dairies, many researchers focus on developing better starter cultures to improve food qualities and functionalities. Sørensen et al. investigated antimicrobial agents that target the cell envelope to improve LAB texturizing abilities in milk fermentation. They created pressure for strain mutagenesis using ampicillin, vancomycin or triclosan, and selected some mutants with improved phenotypic traits in a high-throughput manner based on the liquid handling pressure (Poulsen et al., 2019). The identified genetic changes in the mutants were distributed in genes encoding sugar transporter, ATPase, cell membranes and others.

In addition to the dairy industry, LAB play important roles in plant-food diversification with different functions. As one example, LAB fermentation can improve the bioavailability and bioaccessibility of phenolic compounds that have antioxidant activities. It can add value to the production of newly fermented plant-based foods. Bertsch et al. studied three LAB strains to produce functional bioingredients by single or co-fermentation with yeast. They found LAB fermentation could improve the extraction and production efficiency of both free and bound phenolic compounds. In monoculture, the total and bound phenolic compounds were higher than co-culture. But in co-culture, it had a higher content of water-soluble polysaccharides. LAB fermentation can also increase the bioavailability of micronutrients. Selenium (Se) is an essential micronutrient for most living organisms and Se deficiency is associate with many diseases. Martinez et al. explored the transformation of selenite into selenium-nanoparticles and Se-amino acids using LAB. Some *Lactobacillus* show great potential for the enrichment of fermented foods with selenium.

Due to the GRAS status and their metabolic functions, some LAB exhibit promising health-promoting applications as probiotics or therapeutic agents. Rasinkangas et al. worked on the well-characterized probiotic *L. rhamnosus* GG and generated its non-GMO (genetically modified organisms) mutants using Ethyl methanesulphonate mutagenesis. 13 mutant strains that showed increased mucus-adherent phenotypes were characterized and their whole genomes were sequenced. The identified gene mutations in either pili-encoding genes *spaCBA* or other genes open a window on understanding the synthesis and regulation of pill formation as well as the mucus binding properties. The developed non-GMO derivatives show direct potential in the pharmaceutical industries. Since many *Lactobacillus* can colonize mucosa in humans and animals, the surface display of functional proteins on them could have biomedical applications. Tay et al. tested a new cell-anchoring domain CAD4a bound to peptidoglycan in LAB cell wall and optimized the conditions for its optimal anchoring. They demonstrated that the surface-displayed superoxide dismutase using CAD4a could be protected from gastric digestion in a polymer matrix.

Last but not least, many LAB have been metabolically engineered as cell factories. Bøe and Holo engineered *L. lactis* MG1363 to produce menaquinone (MK, vitamin K2). They found that the key enzymes, including *menF* (isochorismate synthase), *menA* (DHNA polyprenyltransferase), *mvk (*mevalonate kinase) and *preA* (prenyl diphosphate synthase), were able to control the MK flux. The combined overexpression of *preA*, *menA* and *mvk* gave rise to a higher level of MK7-9 (680 nmol/L) than individual overexpression. This work elucidated metabolic bottlenecks in the biosynthetic MK pathway and provided a foundation for further engineering to increase MK production or develop food-grade strains with high vitamin K2 content. In another work, Suo et al. worked on the same strain *L. lactis* MG1363 and rewired the metabolic flux for producing pyruvate through knocking out all the competitive pathways leading to the formation of lactate, acetate, acetoin and ethanol. They further optimized the fermentation medium and achieved a high titer (40 g/L) and high yield (close to 80% of the theoretical maximum) of pyruvate by using dairy waste as substrate. These results demonstrate the economic feasibility of using *L. lactis* for producing valuable products on waste streams.

Additionally, Liu et al. showed the co-production of nisin and phenylacetic acid (PLA) that displayed enhanced antibacterial activities against pathogenic microorganisms, such as *S. xylosus* and *Micrococcus luteus*. They constructed a new *L. lactis* strain based on a nisin-producing strain F44 by knocking out two main L-lactate dehydrogenases (LDH) and expressing a D-LDH mutant. The new strain delivered high antimicrobial activities.

Overall, we believe this collection presents benchmark work in the LAB field. It will benefit the readers to gain the recent developments and the LAB community to move forward for the next achievements.
